# Transcriptome Analysis of the Liver and Muscle Tissues of Dorper and Small-Tailed Han Sheep

**DOI:** 10.3389/fgene.2022.868717

**Published:** 2022-04-11

**Authors:** Hongyang Peng, Mingyue Hu, Zhengxi Liu, Weining Lai, Lulu Shi, Zhongli Zhao, Huihai Ma, Yumei Li, Shouqing Yan

**Affiliations:** ^1^ College of Animal Science, Jilin University, Changchun, China; ^2^ Institute of Animal Husbandry and Veterinary, Jilin Academy of Agricultural Sciences, Gongzhuling, China

**Keywords:** RNA-seq, dorper, small-tailed Han sheep, growth, mutton

## Abstract

It is well known that Dorper (DP) is a full-bodied, fast-growing and high dressing percentage breed, while the production performance of Small-tailed Han sheep (STH) is not so excellent, in contrast to DP. Therefore, in this study, a comparative transcriptomic analysis of liver and muscle tissues from DP and STH breeds was carried out to find differentially expressed genes (DEGs) that affect their growth and meat quality traits. The results showed that the total number of DEGs was 2,188 in the two tissues. There were 950, 160 up-regulated and 1,007, 71 down-regulated genes in the liver and muscle, respectively. Several DEGs such as *TGFB1*, *TGFB3*, *FABP3*, *LPL* may be associated with growth and development in DP. Also, several GO terms were found to be associated with muscle growth and development, such as developmental growth (GO:0048589), and myofibril (GO:0030016). Further validation of eight genes (6 up-regulated, and 2 down-regulated) was performed using quantitative RT-PCR. These findings will provide valuable information for studying growth and development as well as meat quality traits in sheep.

## Introduction

Since sheep were domesticated 8∼11 thousand years ago in Fertile Crescent, they have been providing mankind with excellent products such as meat, wool and milk (Lv et al., 2015). As a country with a large population, China has a huge sheep industry chain with a large consumer of sheep meat. Mutton is becoming increasingly popular with people because of its rich nutrition, high protein and low-fat characteristics. The economic value of a mutton sheep is primarily based on the quantity and quality of the meat. Meanwhile, the growth rate of the sheep is one of the main factors affecting meat yield. Therefore, one breeding objective of mutton sheep is to increase growth rate and meat yield (Seyedsharifi and Hamze Zadeh Azar, 2016). In the same environments, the genetic background becomes one of the important factors influencing meat production (Hegarty et al., 2006; Aali et al., 2017).

The Dorper (DP) is a commercial mutton breed native to South Africa, known for its excellent meat quality and outstanding growth potential (Milne, 2000; Schoeman, 2000). Meanwhile, it is an early maturing and fat depositing breed with a high average daily gain (Brand et al., 2017). Many studies on improving the growth of indigenous breeds by crossing them with Dorper have been reported (Weldeyesus, 2017; Shi et al., 2021). As a remarkable local breed in China, the Small-tailed Han sheep (STH) has a significant characteristic of high fecundity (Wang et al., 1990). Nevertheless, compared to commercial mutton sheep (e.g., Dorper, Dorset and Charollais), STH has a slower growth rate and a lower meat yield (Zhang et al., 2014; Sun et al., 2016), which seriously affects the production efficiency. Differences in the productive performance of DP and STH are mainly determined by genetic material and related regulatory factors. The wide application of RNA-seq technology has accelerated the selection and breeding of livestock. Based on transcriptome comparisons, transcripts in tissues can be well characterized, identified and quantified. Many genes affecting important traits have been identified by RNA-seq (Chen et al., 2011a; Chen et al., 2011b; Ayuso et al., 2015; Weber et al., 2016; Aali et al., 2017). To date, the comparative transcriptome analysis between DP and STH has not been described clearly and accurately.

Therefore, this study was designed to identify genes associated with growth and meat quality in liver and muscle tissues of DP and STH using RNA-seq. It will provide a theoretical basis for improving sheep breeding practices in China and may contribute to the understanding of molecular regulatory mechanisms of sheep production performance.

## Materials and Methods

### Experimental Animals

A total of six male individuals (three of each breed) were randomly selected from purebred SAMM population of Sheep Farm of Jilin Academy of Agricultural Sciences (Changchun, China), which with similar date of birth and weaned at around 45 days, were kept in the same environment (same diet and management practices). The animals were weighed before slaughter and analyzed using the T-test. Two comparison groups were designed for DP and STH in this study, a liver comparison group (DP_L vs. STH_L) and a muscle comparison group (DP_M vs. STH_M), and lambs were slaughtered at an approximate age of 6 months, then liver and Longissimus dorsi tissue (muscle) were collected into the tubes, which were labeled with sample number (DP_L_1∼3, STH_L_1∼3, DP_M_1∼3 and STH_M_1∼3). All samples were snap-frozen in liquid nitrogen and kept at a temperature of −80°C until RNA extraction.

### RNA Isolation, Library Construction, and Sequencing

Total RNA was extracted from 12 samples using an EasyPure RNA kit (TransGen, Beijing, China) according to the manufacturer’s instructions. The purification, concentration and integrity were detected using a NanoPhotometer spectrophotometer (IMPLEN, CA, United States), the Qubit RNA Assay Kit with the Qubit 2.0 Flurometer (Life Technologies, Carlsbad, CA, United States) and the RNA Nano 6000 Assay Kit of the Bioanalyzer 2,100 system (Agilent Technologies, CA, United States), respectively. Sequencing libraries were generated using NEBNext^®^ UltraTM RNA Library Prep Kit for Illumina^®^ (NEB, MA, United States) following the manufacturer’s recommendations. In brief, 12 RNA samples with high quality and concentration were used to construct the transcriptome libraries, and high-throughput sequencing was performed on the Illumina novaseq 6000 platform according to the manufacturer’s instructions, generating reads of 150 bp in length.

### Data Processing

Clean data were obtained by removing reads, which contained adapters or ploy-N, and low quality reads from raw data by using FastQC software (http://www.bioinformatics.babraham.ac.uk/projects/fastqc) powered by the Linux system. At the same time, Q20, Q30 and GC content of the clean data were calculated. Clean reads were then mapped to the reference genome (Oar_rambouillet_v1.0, http://asia.ensembl.org/Ovis_aries_rambouillet/) and annotated transcripts (http://ftp.esembl.org/pub/release-104/gtf/ovis_aries_rambouillet/) of *Ovis aries* by using the Hisat2 software (https://ccb.jhu.edu/software/hisat2/index.shtml) (Kim et al., 2015). The read numbers mapped to each gene were counted using the featureCounts software (Liao et al., 2014).

### Differential Expression Analysis and Functional Annotation

Analysis of differential expressed genes (DEGs) in each group was performed by DESeq2 R package (Love et al., 2014). In order to improve accuracy, the counts calculated by the featureCounts software were filtered out if the counts were less than 5 in each sample. And the false discovery rate (FDR) of each gene in a pair-wise comparison was determined using the Benjamini-Hochberg method. Cutoffs for differential gene expression were selected as *p*-value and FDR <0.05, and log2fold change (log2|FC|) ≥ 1, and PCA was performed on VST values (implemented in DESeq2). To detect biological functions of DEGs, clusterProfiler R package was used to conduct the Gene Ontology (GO) functional enrichment analysis for cellular components (CC), biological processes (BP), and molecular functions (MF) (Yu et al., 2012). KOBAS.i was applied for Kyoto Encyclopedia of Genes and Genomes (KEGG) pathway analysis (http://kobas.cbi.pku.edu.cn/kobas3/) (Bu et al., 2021). A *p*-value of <0.05 (calculated using Fisher’s exact test) was set as the cutoff criteria for the GO and KEGG pathway functional enrichment analysis.

### RT-qPCR Validation

RNA extraction was performed as described above and then converted to first-strand cDNA using TransScript One-step gDNA Removal and cDNA Synthesis SuperMix (TransGen, Beijing, China). The primers used in this study were designed by Primer 5.0 software (Supplementary Table S1) and were synthesized by Sangon Biotech (Shanghai, China). Quantitative Real-time PCR (RT-qPCR) was conducted on a Stratagene Mx3005P qPCR machine (Stratagene) with SYBR Green Technology. The cycling conditions were as follows: one cycle at 94°C for 30 s, 50 two-segment cycles (94°C for 20 s, 60°C for 1 min) and a final dissociation cycle (95°C for 1 min and progressive rise from 55°C to 95°C). Housekeeping gene *ACTB* was used as an internal control for quantitation, and relative expression of mRNA was calculated using 2^−(ΔΔCt)^ method.

## Results

### Animal Weight and Summary of RNA-Seq Data

The average live weight was 48.27 ± 3.18 kg for DP, which was significantly higher than the weight of 40.73 ± 0.98 kg for STH (*p* = 0.03). To obtain a global overview of genes related to sheep growth and meat quality, we performed two pairwise comparisons: DP_L vs. STH_L, DP_M vs. STH_M. Twelve separate cDNA libraries were constructed from the liver and muscle. A total of 590,460,078 raw reads were obtained from liver and muscle tissues, the total number of bases for each of the samples varied from 6.4 to 8.4 Gb. After removing adapters, low-quality and low-complexity reads, high-quality RNA sequencing data were generated. Subsequently, 561,560,544 clean reads (84.23 Gb) were obtained, with each sample having >6.13 Gb. The Q20, Q30 and GC content of the clean data were simultaneously calculated. The Q20 (the percentage of bases with a Phred score greater than 20) and Q30 (the percentage of bases with a Phred score greater than 30) were higher than 94%, and the mean average GC content was 50%. The ratio of reads mapping uniquely to the reference sheep genome was ranged from 67.39% to 86.82% (Table 1). PCA and Spearman correlation plots showed that groups can be clearly separated from each other (Supplementary Figures S1, S2).

**TABLE 1:** Statistical information of RNA-seq data in each sample.

Sample	Total raw reads	Total clean reads	Clean reads Q20 (%)	Clean reads Q30 (%)	GC (%)	Clean reads ratio (%)	Unique mapping ratio (%)
DP_L_1	44,084,658	42,221,830	98.10	94.57	46.95	95.77	71.92
DP_L_2	46,946,546	44,876,206	98.00	94.35	47.08	95.59	67.39
DP_L_3	42,719,936	40,874,176	98.11	94.58	46.09	95.68	68.17
STH_L_1	43,005,282	40,916,010	98.25	94.94	49.07	95.14	85.26
STH_L_2	51,488,250	48,376,738	98.26	94.99	51.10	93.96	86.23
STH_L_3	54,307,070	50,738,118	98.26	95.08	50.93	93.43	80.03
DP_M_1	50,020,826	47,539,300	98.19	94.95	53.79	95.04	84.57
DP_M_2	45,877,652	43,800,592	98.19	94.79	53.70	95.47	85.52
DP_M_3	58,070,778	54,798,726	98.27	95.10	54.02	94.37	86.82
STH_M_1	51,324,266	49,005,802	98.27	94.99	51.38	95.48	85.81
STH_M_2	48,342,334	46,191,230	98.23	95.02	52.73	95.55	84.76
STH_M_3	54,272,480	52,221,816	98.15	94.73	52.67	96.22	84.87

### Differentially Expressed Genes Analyses

**FIGURE 1:**
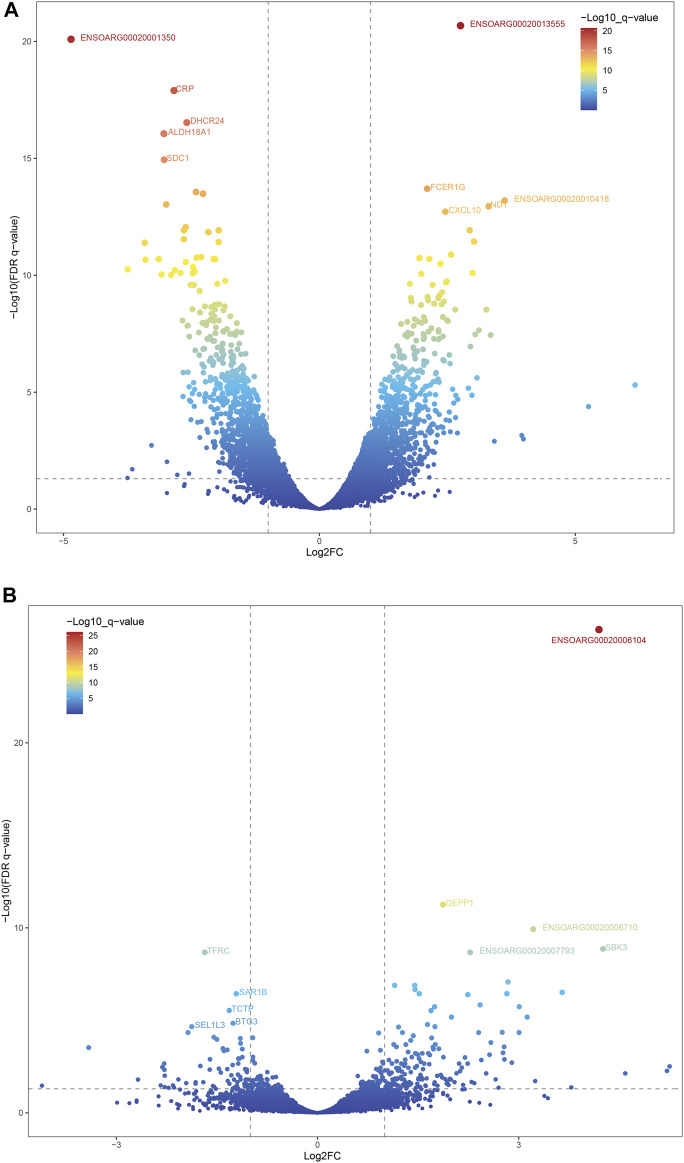
The volcano plots of DEGs of the two groups. Two vertical lines indicated the expression fold change (DP vs. STH) ≥1 and  ≤−1, respectively, and the horizontal line indicated the adjusted *p*-value (FDR *q*-value) of 0.05. The color of the dot represented the FDR (*q*-value) levels. The top 5 DEGs were labeled on the plots. **(A)** DEGs of the liver; **(B)** DEGs of the muscle.

A total of 12,823 and 13,685 genes were found in liver tissues of DP and STH, respectively. Also, 13,061 and 12,899 genes were found in muscle tissues. The intersection analysis revealed that 11,129 genes were expressed in all 4 groups (FPKM > 0) (Supplementary Figure S3A). Then the DESeq2 R package was used to obtain DEGs. A total of 1957 DEGs were found when comparing liver transcriptomes of DP and STH sheep (FDR < 0.05) (Supplementary Table S2). Of these, 950 genes had higher expression in DP sheep compared to STH sheep and are therefore referred to as “up-regulated”, while the remaining 1,007 genes had lower expression in DP sheep and are accordingly named “down-regulated” (Figure 1A). Regarding the muscle transcriptome, 160 and 71 DEGs were detected as up-regulated and down-regulated, respectively (Supplementary Table S3) (Figure 1B). Integration analyses of DEGs revealed that 14 DEGs were up-regulated in both liver and muscle of DP, and correspondingly, the number of down-regulated in both tissues was 12 (Supplementary Figures S3B,C).

### GO and KEGG Pathway Analyses of DEGs

**FIGURE 2:**
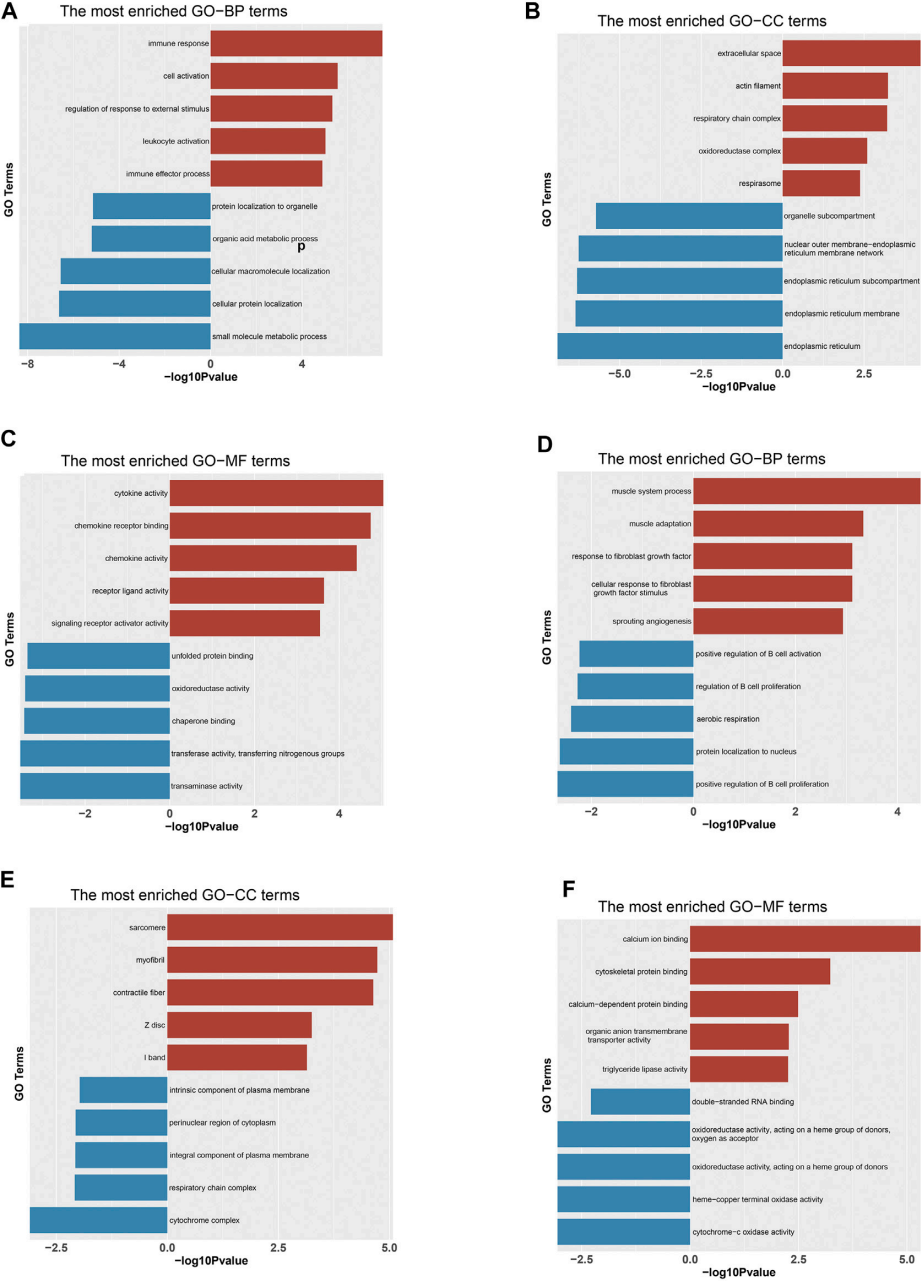
Significantly enriched Gene Ontology (GO) terms of DEGs based on their functions. Bar chart of the top 10 BP **(A)**, CC **(B)** and MF **(C)** terms in the enrichment analysis of DEGs in the liv-er. Bar chart of the top 10 BP **(D)**, CC **(E)** and MF **(F)** terms in the enrichment analysis of DEGs in the muscle. BP: biological process; CC: cellular component; MF: molecular function. “Red” represents for GO terms of up-regulated genes and “Blue” represents for GO terms of down-regulated genes in DP.

GO enrichment analysis was performed to shed light on the potential function of DEGs concerned with growth and fat deposition-related traits between DP and STH. In the study, we separated the up- and down-regulated genes for GO analysis and found that many up-regulated DEGs enriched two GO terms related to growth development: developmental growth (GO:0048589) and positive regulation of developmental process (GO:0051094). And some up-regulated genes were associated with adaptability and immunity, such as adaptive immune response (GO:0002250). Down-regulated genes were enriched to a number of metabolic pathways, including small molecule metabolic process (GO:0044281), carboxylic acid metabolic process (GO:0019752), and oxoacid metabolic process (GO:0043436). Moreover, the term negative regulation of growth (GO:0045926) was discovered. In the muscle group, up-regulated genes were associated with calcium ion binding (GO:0005509), sarcomere (GO:0030017), myofibril (GO:0030016), while down-regulated genes were associated with cytochrome complex (GO:0070069), positive regulation of B cell proliferation (GO:0030890), respirasome (GO:0070469) (*p* < 0.05). The top 10 significant GO terms in each of the three categories were shown in (Figures 2A–F). All GO results were given in Supplementary Tables S4, S5.

**FIGURE 3:**
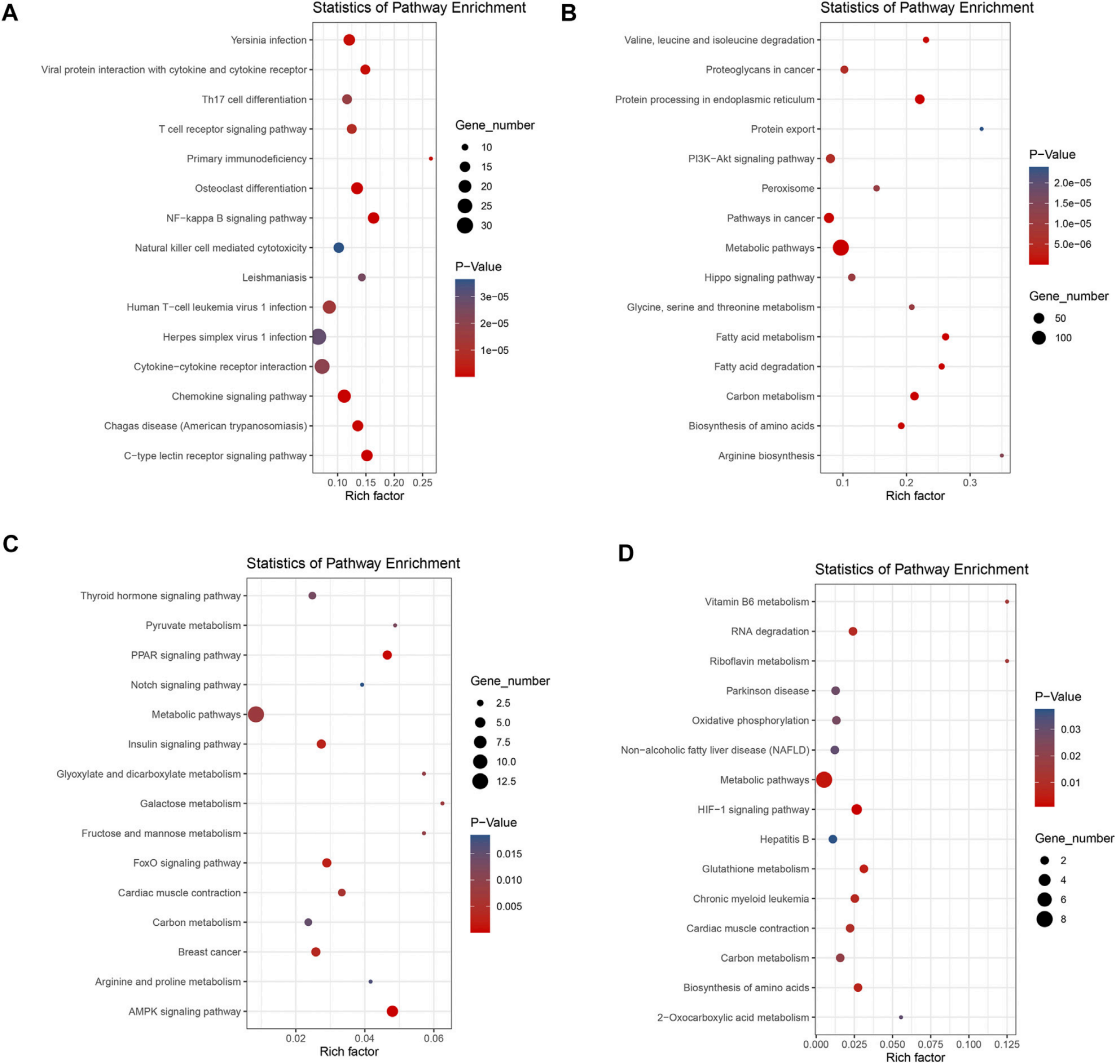
Bubble plots showing functional pathways of KEGG enrichment: The top 15 pathways shown in **(A)** pathways of up-regulated genes of liver; **(B)** pathways of down-regulated genes of liver; **(C)** pathways of up-regulated genes of muscle, and **(D)** pathways of down-regulated genes of muscle. Up- and down-regulation refers to gene expression in DP relative to STH.

Based on the KEGG pathway database, we could systematically analyze gene function and molecular networks in cells. In the current study, those DEGs were assigned to 234 KEGG pathways. Of the 193 KEGG pathways in the liver group, up-regulated DEGs were annotated to development-related pathways such as Osteoclast differentiation (oas04380), immune-related pathways, including C-type lectin receptor signaling pathway (oas04625), Chemokine signaling pathway (oas04062), and T cell receptor signaling pathway (oas04660), etc. The down-regulated DEGs of the liver were mainly enriched in several metabolic pathways including Metabolic pathways (oas01100), Carbon metabolism (oas01200) and Fatty acid degradation (oas00071). In addition, signal transduction pathways such as PI3K-Akt signaling pathway (oas04151) and Hippo signaling pathway (oas04390) were also significantly enriched. A total of 41 KEGG pathways were annotated in the muscle, and the up-regulated genes were mainly associated with important signaling pathways and organismal systems such as AMPK signaling pathway (oas04152), FoxO signaling pathway (oas04068), PPAR signaling pathway (oas03320) and Insulin signaling pathway (oas04910). Meanwhile, down-regulated genes are associated with HIF-1 signaling pathway (oas04066), Metabolic pathways (oas01100) and Biosynthesis of amino acids (oas01230). The top 30 significant pathways (15 for up-regulation, 15 for down-regulation) in each group were shown in (Figures 3A–D), and all the significant KEGG results were shown in Supplementary Tables S6, S7. Statistics for GO and KEGG results for each group were available in Supplementary Table S8.

### RT-qPCR

**FIGURE 4:**
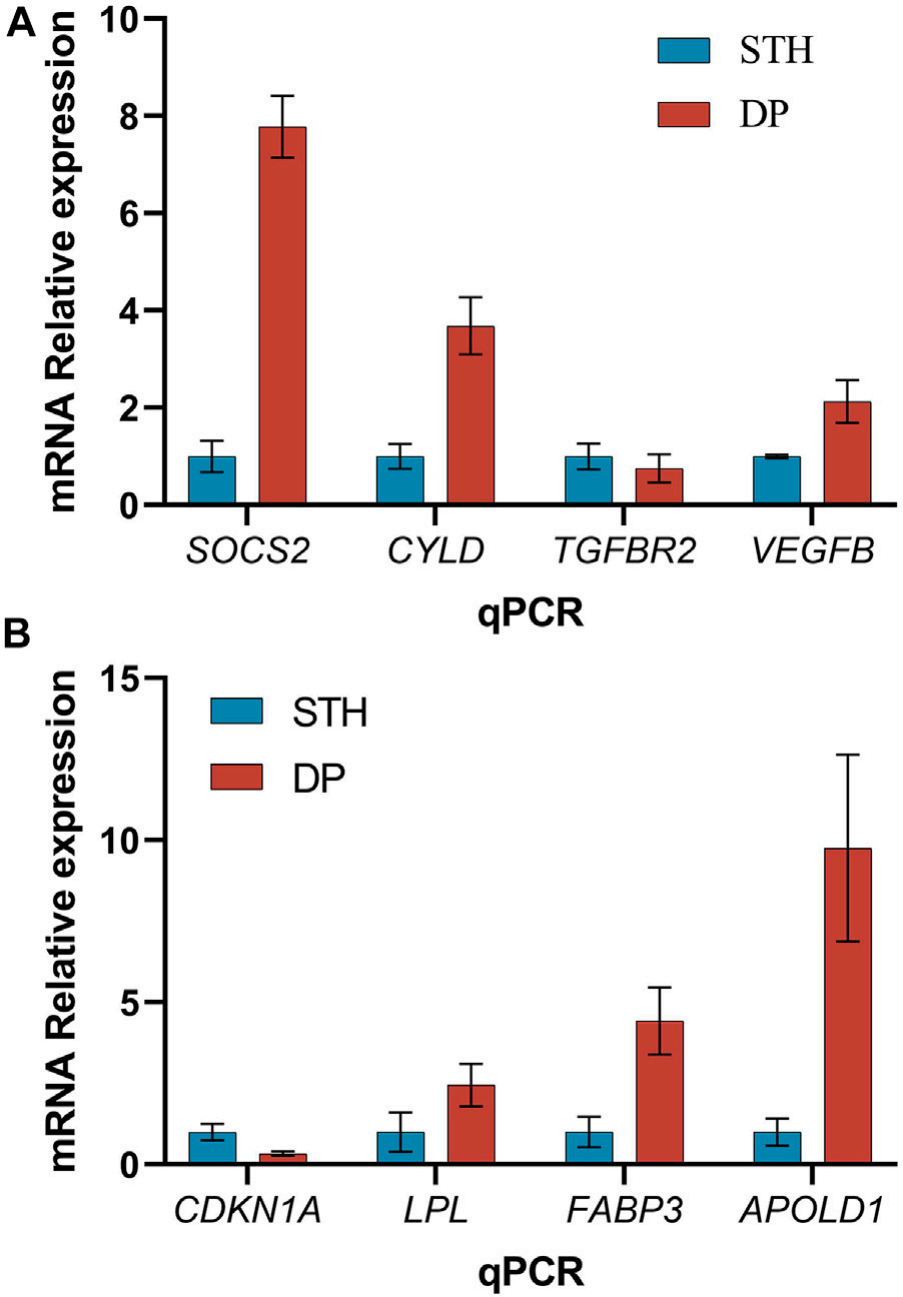
Quantitative real time PCR analysis (mean ± s.d., n = 3 biological replicates). **(A)** Results of 4 DEGs of liver; **(B)** Results of 4 DEGs of muscle.

In order to validate the results observed based on the RNA-seq data, the relative expression of 8 genes was quantiﬁed with RT-qPCR in the 12 samples. Six up-regulated (*SOCS2*, *CYLD*, *VEGFB*, *FABP3*, *LPL*, *APOLD1*) and 2 down-regulated genes (*TGFBR2*, *CDKN1A*) were selected. The validation results showed that the tendency of qPCR data was consistent with that of transcriptome sequencing data (Figures 4A,B).

## Discussion

Mutton is a great source of food with a high content of protein and vitamins, therefore lots of people in the world could be mutton potential consumers. Because of its rapid growth and high meat production, DP is often used as a sire to improve the production performance and carcass quality of local breeds (Cheng et al., 2020). STH is widely reared in northern China due to its high reproductive capacity, imperfectly, in terms of meat production, it is not as good as the imported breeds (Di et al., 2012; Cheng et al., 2020). Many genes related to meat quality, growth and development have been identified in sheep (Di et al., 2012; Sun et al., 2016). Zhang et al. initially reported DEGs in biceps brachii tissues between DP and STH (one sample per breed) (Zhang et al., 2014). Indeed, their study provided very precious information of the DP and STH muscle transcriptomes, unfortunately, the information is limited, as a result of two individuals and inaccurate annotation of the selected reference genome. Consequently, an initial objective of this study was to identify the DEGs of the liver and muscle tissues, which may have an important role in the growth and meat yield of sheep.

In the liver, transcriptome analysis revealed that gene expression of DP and STH was significantly different. Among the DEGs, we found two GO terms namely developmental growth (GO:0048589) and positive regulation of developmental process (GO:0051094). The two terms contain many genes, which are associated with growth and development in livestock or poultry. For example, *Hesx1* polymorphism is associated with average daily gain in bovine (Lai et al., 2009), *MUSTN1* is thought to be involved in muscle growth and development in chickens (Li et al., 2013), and *ATP2B1* is associated with growth and meat quality in pigs (Ramos et al., 2009). More importantly, we identified two members of the transforming growth factor-beta, *TGFB1* and *TGFB3*. *TGFB1* has been shown to be associated with muscle growth and pig feed utilization, and *TGFB3* plays an important role in the growth and production (Long et al., 2003; Jing et al., 2015; Cao et al., 2017). SOCS2 belongs to the suppressors of cytokine signaling family, which has a central SH2 domain and a C-terminal SOCS box. The proteins of SOCS2 are multifunctional through the JAK/STAT/SOCS pathway (Metcalf et al., 2000), as modulators of cytokine and growth factor signaling. SOCS2 acts in the growth hormone (GH) signaling pathway and is therefore associated with cell growth (Metcalf et al., 2000; Horvat and Medrano, 2001; Greenhalgh et al., 2005). Low levels of *SOCS2* expression inhibit several signaling pathways, including GH, prolactin, and interleukins, which negatively regulate growth hormone and insulin-like growth factor-1, whereas high levels of *SOCS2* can restore or even increase the responsiveness of these growth factors (Favre et al., 1999; Pezet et al., 1999; Tannahill et al., 2005; Piessevaux et al., 2006). Further, the SOCS2 p.R96C mutation in sheep resulted in better traits such as body weight, body size, and milk production (Rupp et al., 2015). In addition, some up-regulated DEGs were significantly enriched in several immune-related terms and KEGG pathways, which makes sense as adaptability to the environment is one of the characteristics of Dorper. In our study, these genes were significantly more highly expressed in DP than in STH, and it is reasonable to assume that they have contributed to the growth of DP.

In the muscle, we identified a subset of up-regulated genes (for example, *FABP3*, *LPL*), which are probably involved in adipogenesis and fat deposition. FABP3 belongs to the fatty acid-binding proteins (FABPs) family, which, like other members of the FABPs family, is an intracellular protein involved in the transport of fatty acids from the plasma membrane to sites of ß-oxidation and triacylglycerol or phospholipid synthesis, regulating cell growth and proliferation (Chmurzyńska, 2006; Jurie et al., 2007) Many studies have shown that the *FABP3* gene is involved in muscle development and meat marbling (Uemoto et al., 2007; Arora et al., 2014). Xu et al. (2018) found that *FABP3* was associated with fat deposition in Yorkshire pigs. Similarly, Xu et al. (2020) reported that the expression of the *FABP3* had a positive effect on the intramuscular fat (IMF) content in different muscles of Tan sheep. Lipoprotein lipase (*LPL*), which regulates fat in muscle and thus affects carcass quality, has been shown to significantly affect the fatty acid composition of Korean cattle muscle (Oh et al., 2013). Furthermore, both *LPL* and *FABP3* are enriched in the PPAR signaling pathway, which is known to be a key regulatory pathway in adipogenesis (Lehrke and Lazar, 2005). In addition to this, we also found up-regulated genes including *MYL2*, *MYL6* and *TNNC1*, which have been shown to correlate with meat quality (PilNam et al., 2008; Pierzchala et al., 2014; Muniz et al., 2020). In this experiment, *FABP3* and *LPL* were significantly up-regulated in the muscle tissue of the DP, this is consistent with the actual situation, as fat deposition of DP is early (Brand et al., 2017). Hence, the increased expression level of the *FABP3* and *LPL* may contribute to intramuscular fat deposition in Dorper.

In this study, 14 up- and 12 down-regulated DEGs were identified in both tissues of DP and STH, respectively. Among the up-regulated genes, the *BICC1* gene is associated with human skeletal muscle hypertrophy and is involved in skeletal cell differentiation (Mesner et al., 2014; Seaborne et al., 2018), Moreover, this gene has been revealed as one of the selection signals for body size, meat, and growth-related trait, as well as, has been associated with birth weight in sheep (Almasi et al., 2020; Li et al., 2020), Another up-regulated gene, *TFAP4* was considered to be related to chest girth in genome-wide association studies (Tao et al., 2020). These genes were significantly higher in both muscle and liver of DPs than in STH, suggesting that they are likely to be involved in body weight at slaughter in DP. Of the 12 co-expressed down-regulated genes, *TFRC* and *MAPK6* are thought to be associated with immunity and mastitis, *LARP1B* and *ODC1* are associated with reproductive traits, such as oestrogen and sperm quality (Park et al., 2016; Nasser et al., 2020; Zhao et al., 2020; Ghahramani et al., 2021; Zhong et al., 2021). In addition, considering that the *CDKN1A* gene has a negative growth regulatory role as showed in the GO enrichment analysis, the down-regulated expression of these genes may be related to the highly reproductive request, slow growth and developmental characteristics of the STH breed. However, more studies are necessary for a better understanding of the molecular networks and genes regulating economically important traits in sheep.

## Conclusion

In this study, comparative transcriptomic analysis was applied to the liver and muscle of Dorper and Small-tailed Han sheep. Some DEGs, such as *TGFB1*, *TGFB3*, *FABP3*, *LPL*, and a number of GO terms may be associated with growth and fat deposition in Dorper. In addition, the *CDKN1A* gene was identified, which may be involved in the growth-regulating role of Small-tailed Han. The accuracy of the RNA-seq was verified using RT-qPCR. The mechanisms behind these differences need to be further investigated. Even so, we expect that our analysis will provide accessible information for future research.

## Data Availability

The datasets presented in this study can be found in online repositories. Accession No. PRJNA799452. https://www.ncbi.nlm.nih.gov/bioproject/PRJNA799452/.
