# Supplementary material for: Transcriptome Analysis of the Liver and Muscle Tissues of Dorper and Small-Tailed Han Sheep
Source: Front Genet. 2022 Apr 11;13:868717. doi: 10.3389/fgene.2022.868717 (PMC9035493; doi:10.3389/fgene.2022.868717)
Supplement: Supplementary file 1 [file DataSheet1.ZIP › Supplementary File_V2.docx]

Supplementary File

| 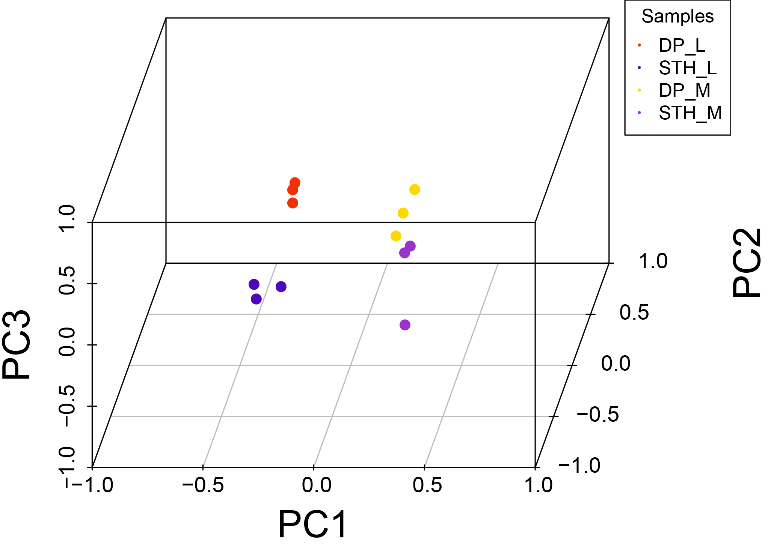 |
| --- |
| **Figure S1** Principal component analysis (PCA) of the RNA-seq samples.  DP_L:the liver of Dorper;DP_M:the muscle of Dorper;STH_L:the liver of Small tailed-han sheep;STH_M:the muscle of Small tailed-han sheep. |
| 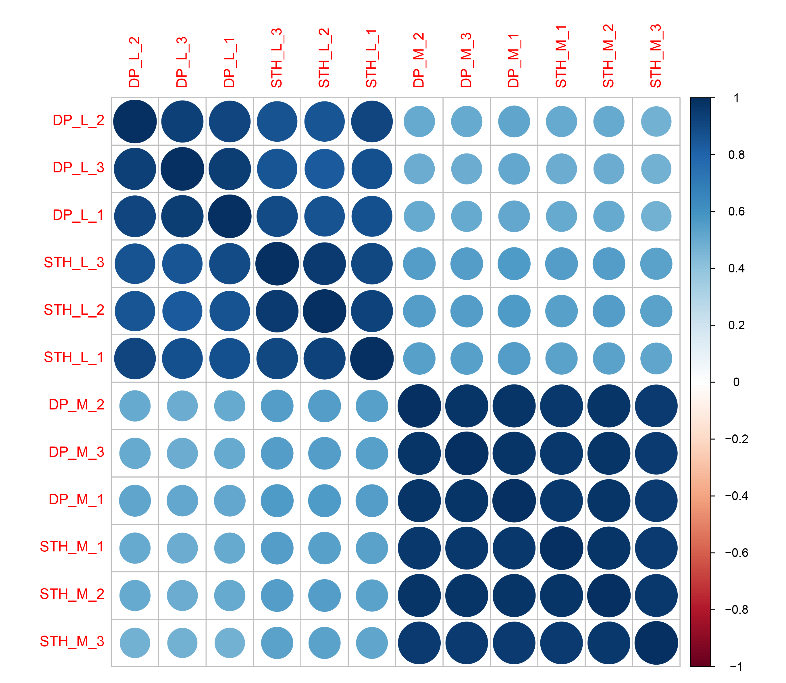 |
| **Figure S2** Sample correlation matrix of the Pearson correlation coefficient of samples.  Abbreviations are the same as above, with the serial number representing the sample number |

| 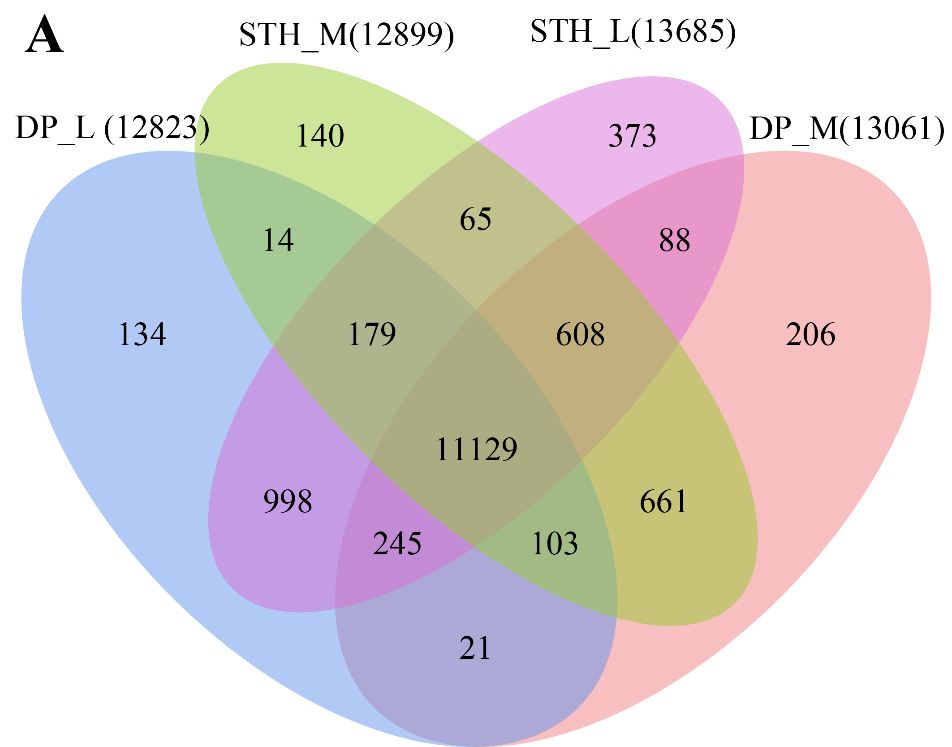 | |
| --- | --- |
| 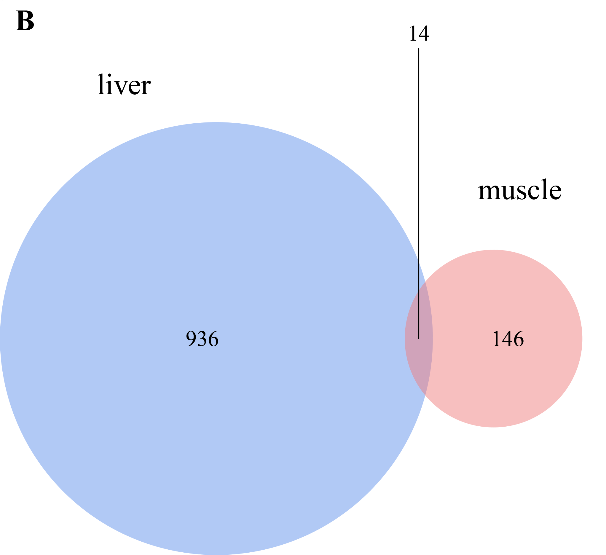 | 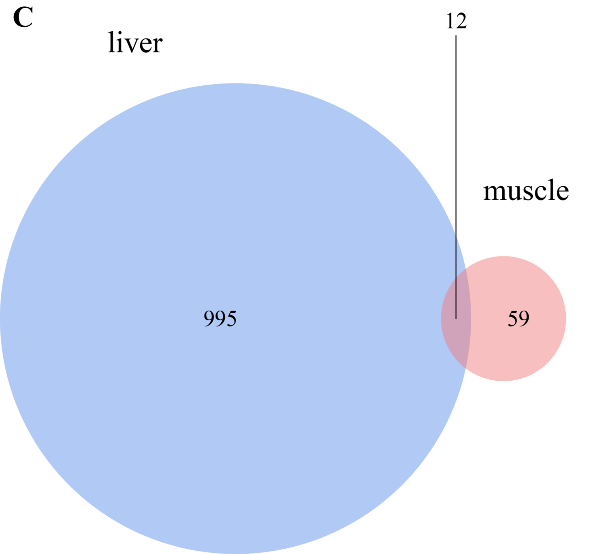 |
| **Figures S3** Venn diagram. (**A**) Venn diagram for the number of genes expressed in liver and muscle of DP and STH (**B**) Venn diagram of up-regulated genes of liver and muscle; (**C**) Venn diagram of down-regulated genes of liver and muscle | |

**Tables S8** Statistics of GO and KEGG results

| terms | number of GO terms | | | number of KEGG |
| --- | --- | --- | --- | --- |
|  | BP | CC | MF |  |
| DP_L | 250 | 23 | 35 | 67 |
| STH_L | 193 | 75 | 35 | 126 |
| DP_M | 138 | 10 | 15 | 26 |
| STH_M | 40 | 7 | 16 | 15 |
