# Supplementary material for: Transcriptome Analysis of the Liver and Muscle Tissues of Dorper and Small-Tailed Han Sheep
Source: Front Genet. 2022 Apr 11;13:868717. doi: 10.3389/fgene.2022.868717 (PMC9035493; doi:10.3389/fgene.2022.868717)
Supplement: Supplementary file 1 [file DataSheet1.ZIP › Supplementary File_V2.pdf]

Supplementary File

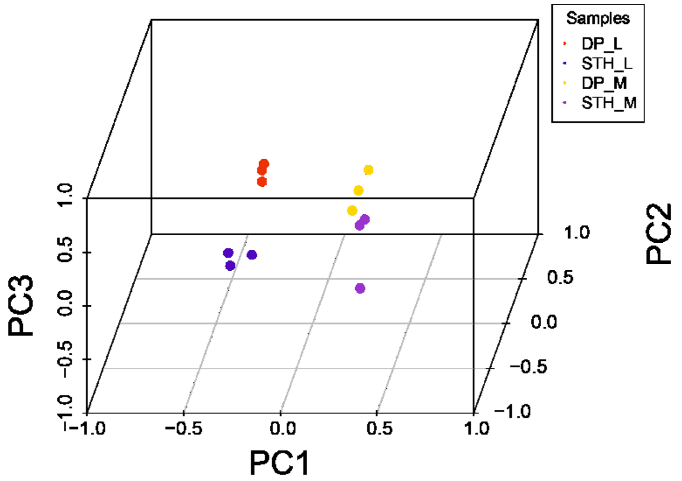

Figure S1 Principal component analysis (PCA) of the RNA-seq samples. DP_L:the liver of Dorper;DP_M:the muscle of Dorper;STH_L:the liver of Small tailed-han sheep;STH_M:the muscle of Small tailed-han sheep.

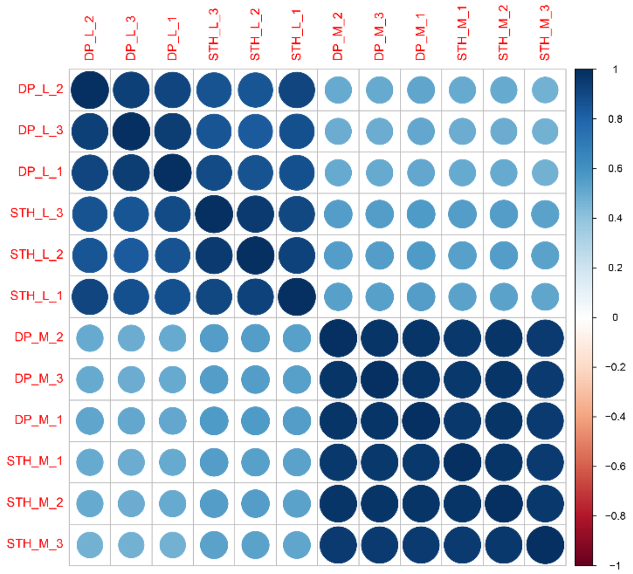

Figure S2 Sample correlation matrix of the Pearson correlation coefficient of samples. Abbreviations are the same as above, with the serial number representing the sample number

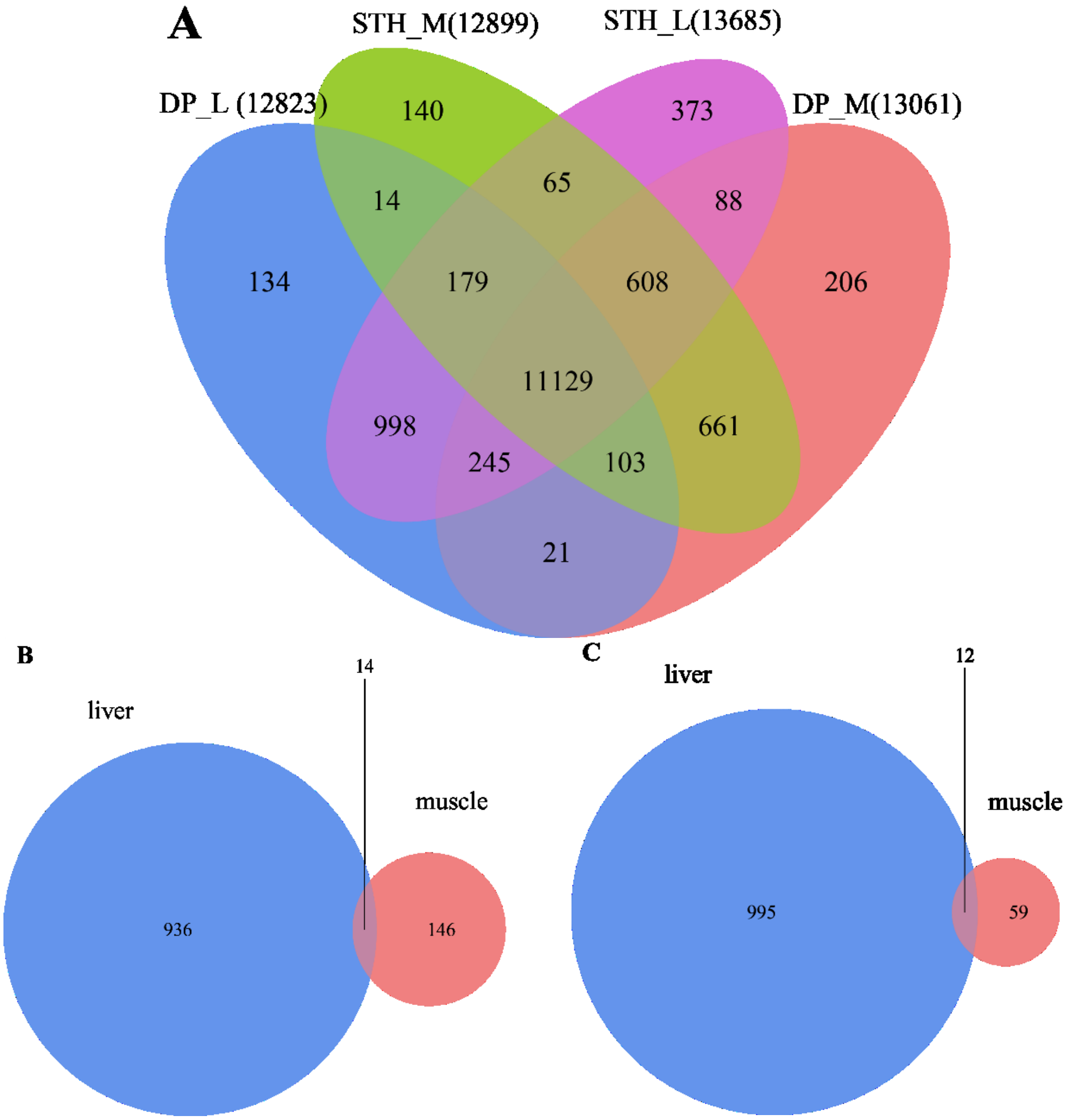

Figures S3 Venn diagram. (**A**) Venn diagram for the number of genes expressed in liver and muscle of DP and STH (**B**) Venn diagram of up-regulated genes of liver and muscle; (**C**) Venn diagram of down-regulated genes of liver and muscle

Tables S8 Statistics of GO and KEGG results

terms	number of GO terms			number of KEGG
	BP	CC	MF	
DP_L	250	23	35	67
STH_L	193	75	35	126
DP_M	138	10	15	26
STH_M	40	7	16	15
