# Supplementary figures and images for: Transcriptome Analysis of the Liver and Muscle Tissues of Dorper and Small-Tailed Han Sheep
Source: Front Genet. 2022 Apr 11;13:868717. doi: 10.3389/fgene.2022.868717 (PMC9035493; doi:10.3389/fgene.2022.868717)

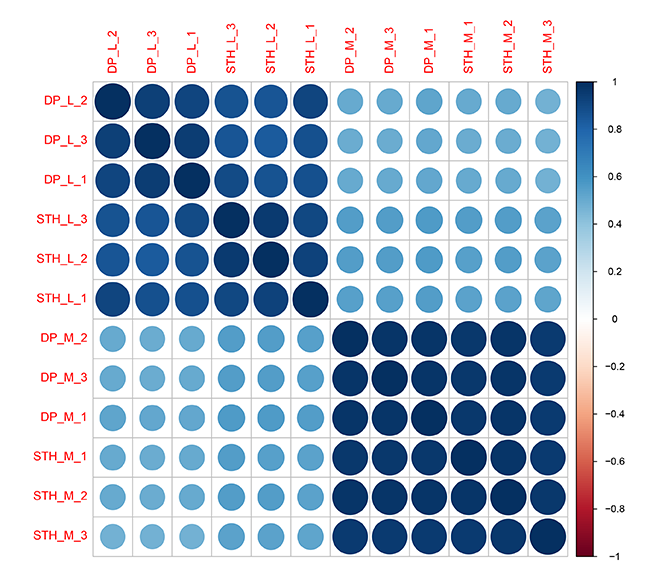

Supplement: Supplementary file 1 [file DataSheet1.ZIP › Figure S1.tif]

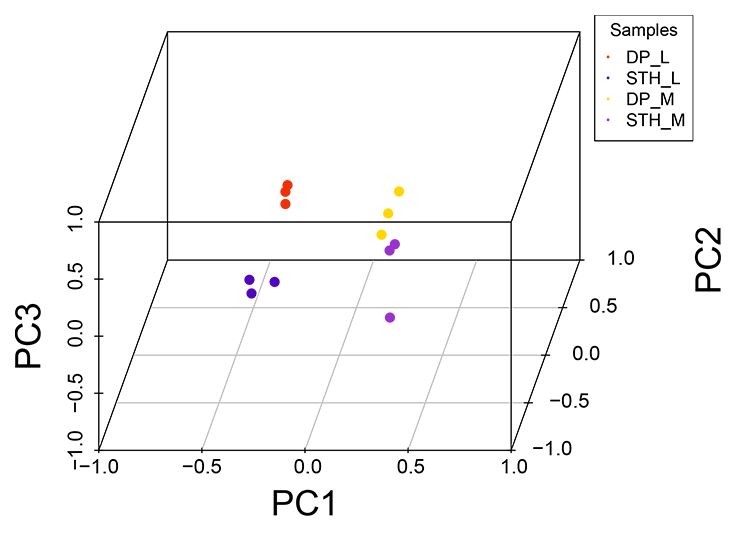

Supplement: Supplementary file 1 [file DataSheet1.ZIP › Figure S2.tif]

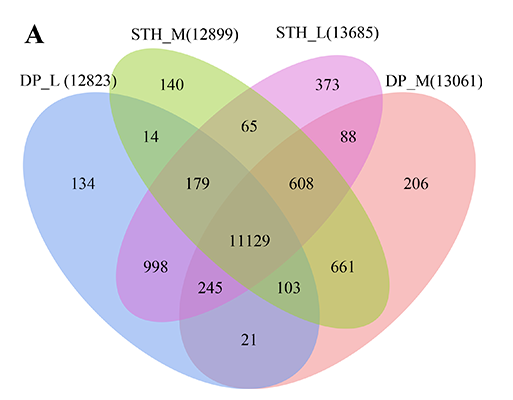

Supplement: Supplementary file 1 [file DataSheet1.ZIP › Figure S3A.tif]

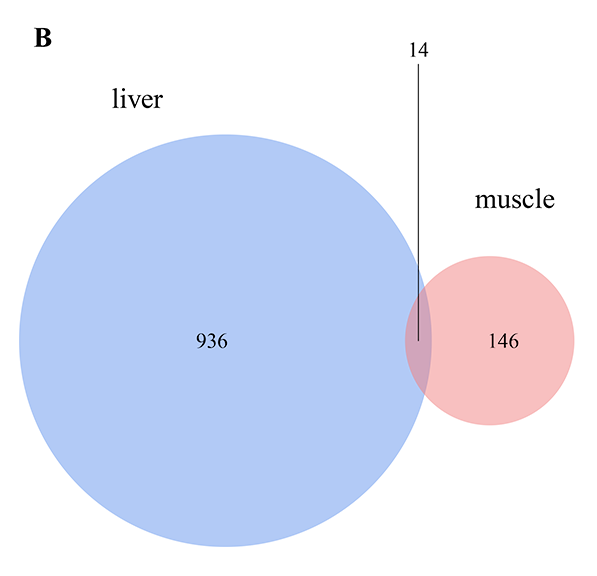

Supplement: Supplementary file 1 [file DataSheet1.ZIP › Figure S3B.tif]

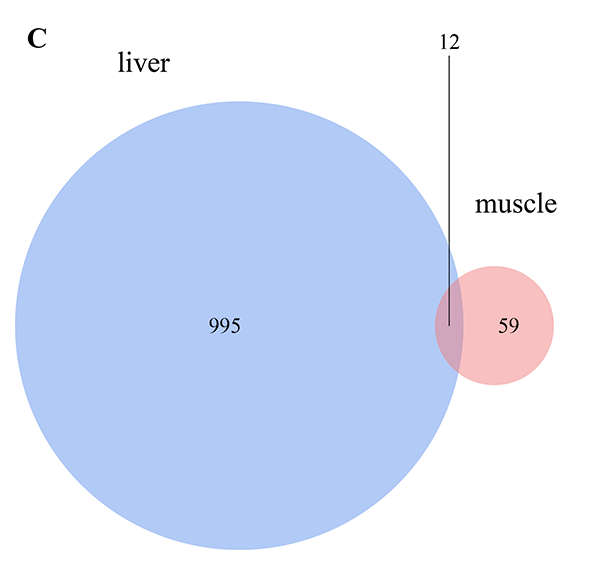

Supplement: Supplementary file 1 [file DataSheet1.ZIP › Figure S3C.tif]
